# Novel *PKD1* Mutation (c.G10086T) Drives High Intracranial Aneurysm Risk in Autosomal Dominant Polycystic Kidney Disease

**DOI:** 10.1111/ene.70086

**Published:** 2025-02-20

**Authors:** Lili Gao, Min Lin, Chenghan Wu, Yuansheng Liao, Zuopeng Lin, Xiaohua Yan, Sheng Lin, Yinzhou Wang, Jing Chen, Zhaocong Zheng, Jushan Lin, Sheng Zhang, Jianhua Guan, Yan Qiu, Jilian Liao, Lihua Wu

**Affiliations:** ^1^ Department of Neurology Second Affiliated Clinical College of Fujian University of Traditional Chinese Medicine Fuzhou China; ^2^ Wuhan Kindstar Clinical Diagnostic Co., Kindstar Globalgene Technology, Inc Wuhan China; ^3^ Department of Neurology and Traditional Chinese Medicine, Fujian Provincial Hospital Shengli Clinical Medical College of Fujian Medical University Fuzhou China; ^4^ Department of Neurosurgery 900th Hospital of PLA Joint Logistics Team Fuzhou China

**Keywords:** ADPKD, Chinese population, genetic screening, intracranial aneurysm, *PKD1* mutation, polycystin‐1, risk stratification, vascular complications

## Abstract

**Background:**

Autosomal dominant polycystic kidney disease (ADPKD) is frequently complicated by intracranial aneurysms (IAs). However, the genetic factors driving the elevated IA risk in ADPKD remain poorly understood. In this study, we identified a novel *PKD1* mutation associated with a remarkably high IA incidence in a large Chinese ADPKD family.

**Methods:**

We conducted whole‐exome sequencing in a three‐generation Chinese ADPKD family (*n* = 24) characterized by an unusually high IA prevalence. The pathogenicity of the identified *PKD1* variant was validated through comprehensive functional studies, including protein localization, calcium signaling, and endothelial cell behavior analyses.

**Results:**

We discovered a novel *PKD1* mutation (c.G10086T) that co‐segregated with disease in all affected family members. Notably, 38.1% (8/21) of the mutation carriers developed IAs, a significantly higher rate than reported in general ADPKD populations (4%–11.5%). Functional studies revealed that this mutation disrupted polycystin‐1 trafficking and impaired calcium signaling, leading to endothelial dysfunction. In vitro experiments demonstrated enhanced angiogenic potential and compromised vascular integrity in cells expressing mutant *PKD1*.

**Conclusions:**

The newly identified *PKD1*:c.G10086T mutation represents a high‐risk genetic variant for IA development in ADPKD. Our findings provide new insights into the vascular complications of ADPKD and suggest that *PKD1* genotyping may help identify patients requiring intensive IA surveillance. This study supports the development of mutation‐specific screening strategies for ADPKD‐associated vascular complications.

## Graphical abstract

The *PKD1* c.G10086T mutation has been validated as a contributor to the genetic heterogeneity observed in polycystic kidney disease patients with intracranial aneurysms through the application of multi‐omics approaches, cellular functional assays, and gene editing techniques.

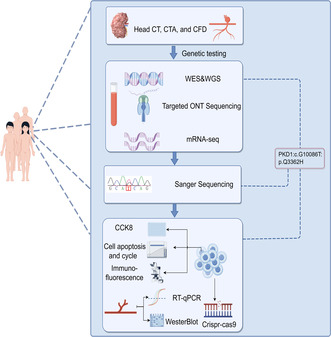

## 1 Introduction

Autosomal dominant polycystic kidney disease (ADPKD) is one of the most common inherited kidney disorders, affecting 1:1000 to 1:2500 individuals worldwide [1, 2, 3]. While renal manifestations are predominant, ADPKD is a systemic disorder with significant vascular complications, among which intracranial aneurysms (IAs) represent a life‐threatening condition [4, 5, 6]. The prevalence of IAs in ADPKD patients (4%–11.5%) is approximately five times higher than in the general population, with a median age of rupture 10 years earlier [7, 8, 9].

Despite the established genetic basis of ADPKD, primarily involving mutations in PKD1 (~78%) or PKD2 (~15%) genes [10, 11, 12], the molecular mechanisms underlying the increased susceptibility to IA formation remain poorly understood [13, 14, 15]. Recent studies have suggested that specific PKD1 mutations might correlate with distinct vascular phenotypes [16, 17, 18], but comprehensive genotype–phenotype correlations for IA risk are lacking [19, 20, 21]. This knowledge gap hampers the development of personalized screening strategies, particularly for populations with different genetic backgrounds [22, 23].

We identified a large Chinese ADPKD family (*n* = 24) exhibiting an unusually high prevalence of IAs. This unique familial clustering suggested a potential genetic driver for enhanced IA susceptibility. Through comprehensive genetic analysis incorporating whole‐exome sequencing, whole‐genome sequencing, and functional validation, we aimed to identify and characterize the genetic variant responsible for the high IA incidence in this family [18, 24].

## 2 Materials and Methods

### 2.1 Study Design and Patient Recruitment

Between January 2017 and December 2022, we conducted a family‐based genetic study at the Department of Neurology, Second Affiliated Clinical College of Fujian University of Traditional Chinese Medicine. The study was approved by the institutional ethics committee (Approval number: SPHFJP‐T2022004‐02) and conducted in accordance with the Declaration of Helsinki. Written informed consent was obtained from all participants.

### 2.2 Clinical Assessment

**FIGURE 1:**
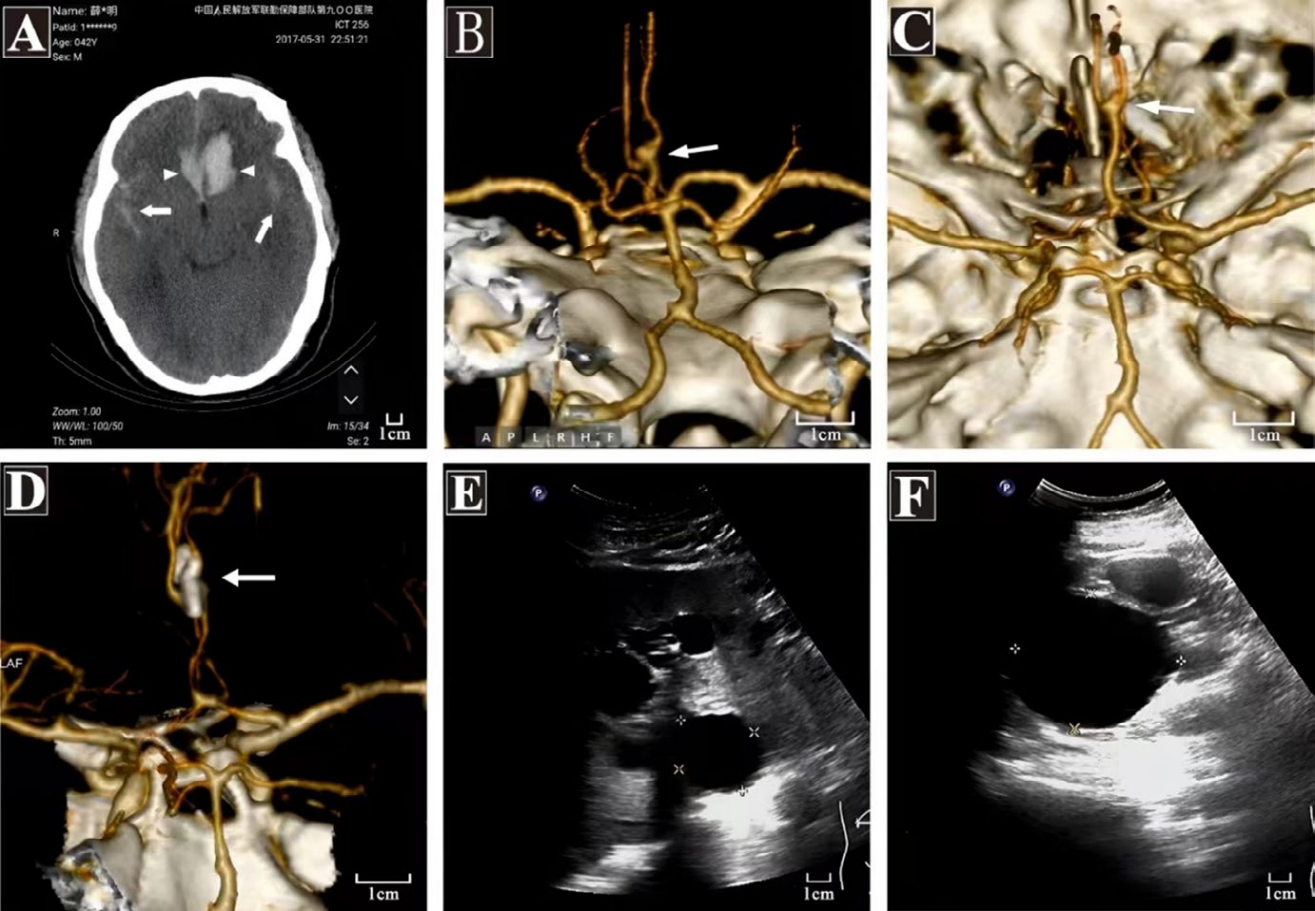
Head computed tomography (CT), CT Angiography (CTA), and abdomen color Doppler ultrasound of proband II:7. (A) Head CT scan showing Fisher grade III subarachnoid hemorrhage with a prefrontal lobe cerebral hematoma (white short arrow). The bleeding area is 3.2 × 2.8 cm, and it is located in the anterior interhemispheric cistern, interpeduncular cistern and bilateral sylvian cistern. High‐density shadow filling (long white arrow) was observed in all cases. (B and C) Preoperative CTA showed a fusiform aneurysm (white arrow) in the A2 segment of the left anterior cerebral artery, approximately 1.1 × 0.8 mm in size. (D) Postoperative review CTA demonstrating correct placement of the titanium clip at the aneurysm site. (E) Color Doppler ultrasound of the left kidney cyst. (F) Color Doppler ultrasound liver cyst.

**FIGURE 2:**
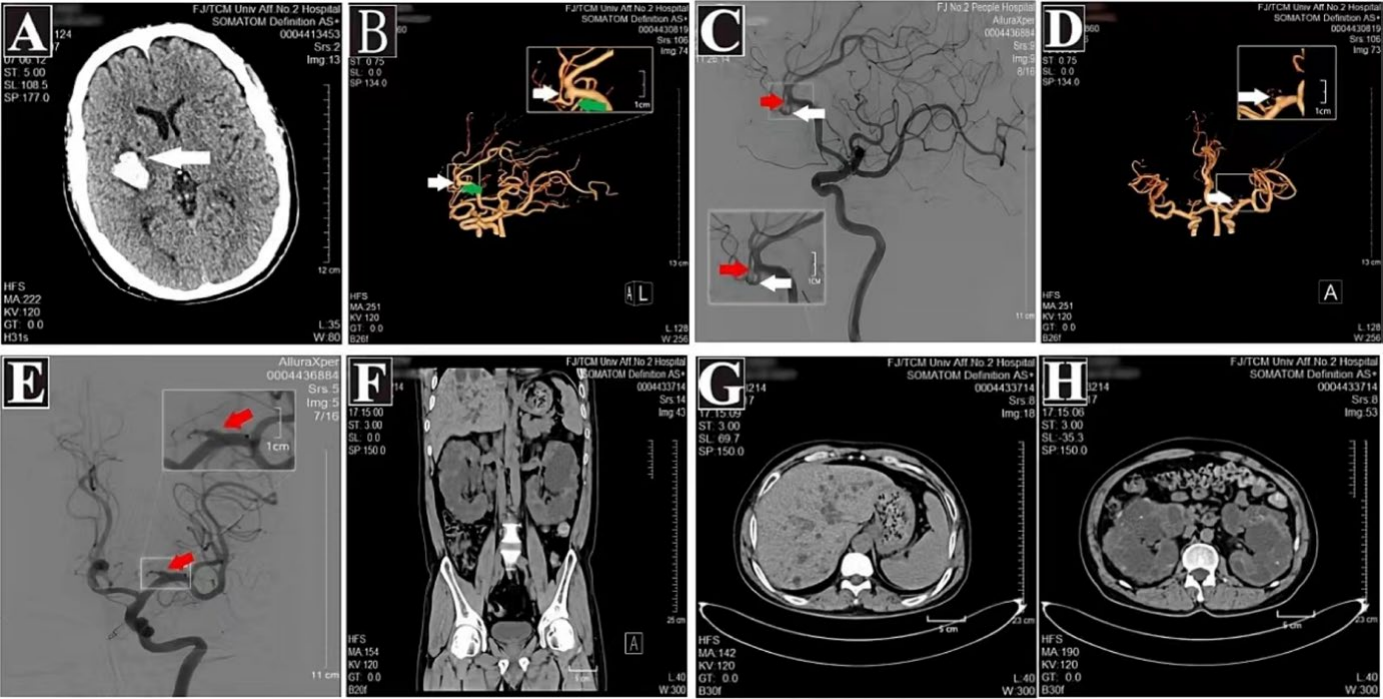
CT and CTA and digital subtraction angiography (DSA) of the second brother of proband II:4. (A) Cerebral hemorrhage in the right basal ganglia area; (B) CTA white arrow indicates anterior cerebral artery aneurysm, the green arrow indicates the callosal artery emerging from the side wall of the aneurysm; (C) DSA, the red arrow indicates anterior cerebral artery aneurysm, and the white arrow indicates the callosal artery emerging from the side wall of the aneurysm; (D) CTA, the white arrow indicates a middle cerebral artery aneurysm; (E) DSA, red arrow indicates a middle cerebral artery aneurysm; (F) Abdominal CT showing a polycystic liver and polycystic kidney; (G) Axial CT showing a polycystic liver; (H) Axial CT reveals polycystic kidney disease.

**FIGURE 3:**
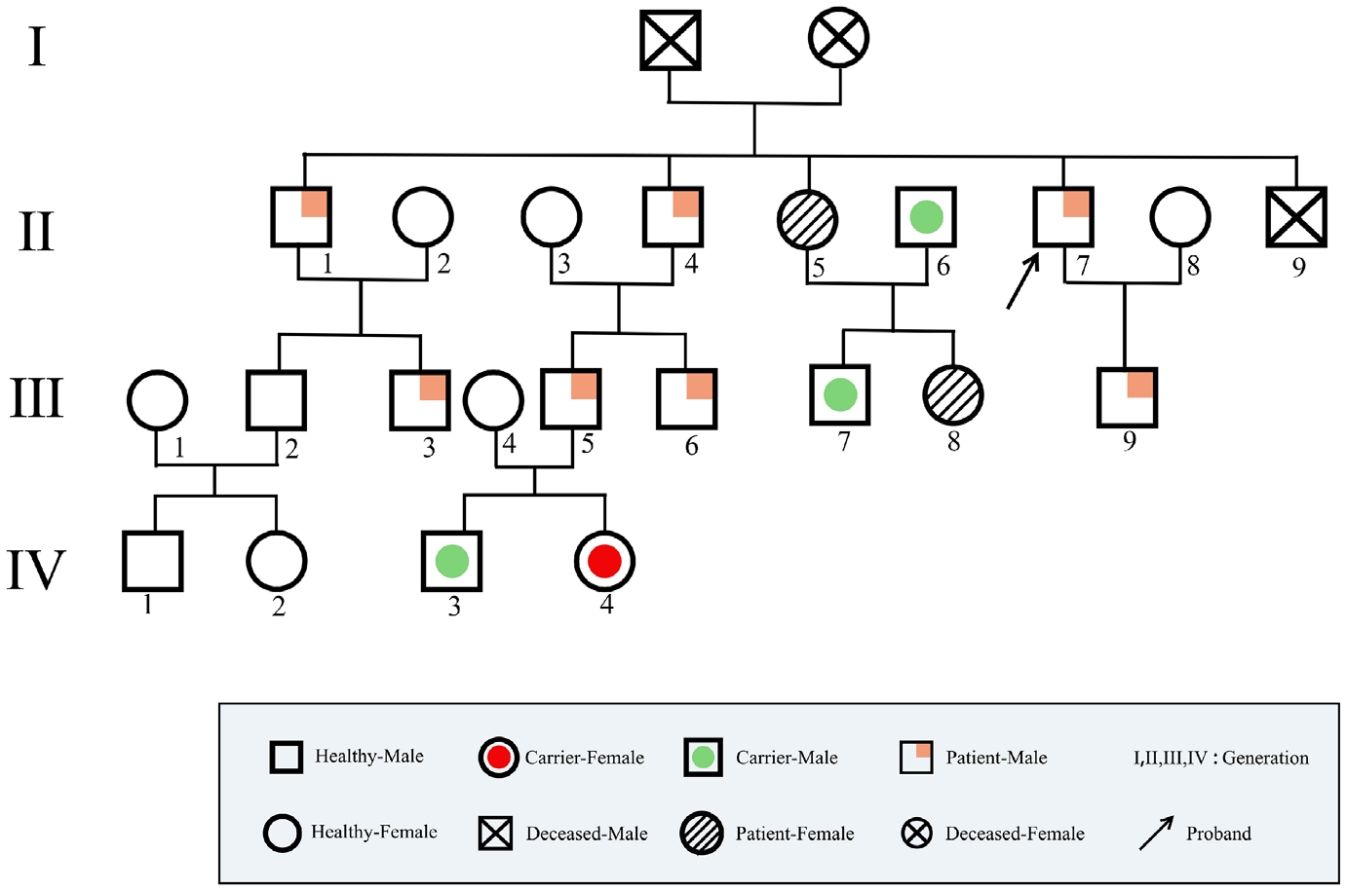
Family genetic profile of the patient.

Patients with ADPKD [25] and IA [26] were recruited from a single Han Chinese family between 2017 and 2022. The study, conducted across the Department of Neurology at the Second Affiliated Clinical College of Fujian University of Traditional Chinese Medicine and the Department of Neurosurgery at the 900th Hospital of PLA Joint Logistics Team, comprehensively examined a four‐generation family cohort. Following initial screening, 21 participants were enrolled in the study, with 3 members excluded due to mortality. Of the 24 family members, 18 were blood relatives and 6 were spouses, with a detailed clinical and genetic investigation revealing the complex inheritance pattern of this rare genetic disorder. The onset imaging data for the proband II:7 and his second brother II:4 are shown in Figures 1 and 2, respectively. A genetic map is shown in Figure 3 (see Supporting Information S1). Peripheral venous blood samples (4 mL) were collected from each participant and tissue samples from small blood vessels in the medial leg of the lower extremities were collected from the proband, the second brother, and a healthy control. These samples were stored at −80°C for centralized detection. Clinical evaluation included the following:

- Detailed medical history and physical examination.
- Laboratory tests (renal/liver function, blood count).
- BrainCT, CTA, MRI, MRA, and Abdominal ultrasound Doppler.
- Cardiovascular assessment.

### 2.3 Genetic Analysis

#### 2.3.1 DNA Extraction and Sequencing

Genomic DNA was extracted from peripheral blood using standard protocols [27]. We implemented a comprehensive genetic analysis workflow incorporating:

- Whole‐exome sequencing (WES).
- Whole‐genome sequencing (WGS).
- RNA sequencing.
- Targeted Oxford Nanopore sequencing.

Libraries were prepared using the Agilent SureSelect Human All Exon V7 kit and sequenced on an Illumina NovaSeq 6000 platform [28].

### 2.4 Variant Analysis

Raw data were processed using the following:

- BWA (v0.7.17) for alignment.
- GATK (v4.1.1.0) for variant calling [29].
- ANNOVAR (v201804) for annotation [30].
- ACMG guidelines for variant classification [31].

### 2.5 Functional Studies

#### 2.5.1 Cell Culture and Gene Editing

HEK293T cells were cultured following standard protocols [32]. The *PKD1*:c. G10086T mutation was generated using CRISPR/Cas9‐mediated gene editing [33].

### 2.6 Protein Analysis

- Western blot analysis using validated antibodies [34].
- Immunofluorescence microscopy [35].
- Cell proliferation and apoptosis assays.
- Migration and invasion assays (see Supporting Information S1).

### 2.7 Statistical Analysis

Data were analyzed using R (version 4.1.0). Continuous variables were compared using Student's *t*‐test or Mann–Whitney *U*‐test. Categorical variables were analyzed using Fisher's exact test. *p* < 0.05 was considered statistically significant [36].

## 3 Results

Clinical characteristics of a polycystic kidney disease family.

A 24‐member family (13 males, 11 females) was investigated, with three unexplained deaths among 21 survivors. Of the 15 blood‐related members.

Genetic Profile:

- Twelve carried PKD1 gene mutations.
- One third‐generation and 2 fourth‐generation members were mutation‐negative.

Clinical Manifestations:

- Ten showed polycystic kidney disease, polycystic liver disease, and splenomegaly.
- Six had intracranial aneurysms (IA).
- Three experienced aneurysm rupture.

Unique Finding:

- One non‐blood‐related spouse harbored a PKD1 mutation and polycystic kidney disease.

Detailed disease status of 18 blood‐related members is presented in Table 1.

**TABLE 1:** Disease incidence in 18 blood‐related family members.

Genetic number	*PKD1*	Onset time	Age of onset	Sex	Symptoms	BP at onset (mmHg)	CTA/DSA	Diagn osis	Color Doppler ultrasound	Outcome
I:1		1997	61	M	Headache	NA	NA	NA	NA	Death
I:2		1977	31	F	Headache dizziness vomiting	NA	NA	NA	NA	Death
II:1	+	2022	55	M	*N*	Normal	Normal	Splenomegaly	Splenomegaly	Survival
II:4	+	2021	53	M	Headache left limb	155/110	IA	ICH	Multiple cysts of liver and kidney	Survival
II:5	+	2009	50	F	Headache dizziness vomiting	202/100	IA	SAH	Multiple cysts of liver and kidney	Hemodialysis
II:9		1996	1	M	NA	NA	NA	NA	NA	Death
II:7	+	2017	47	M	Headache dizziness vomiting	195/89	IA	SAH	Multiple hepatic and renal cysts with stones	Survival
III:1	_	2022	32	M	*N*	Normal	Normal	*N*	Normal	Survival
III:3	+	2022	17	M	*N*	Normal	Normal	PKD	Polycystic kidney	Survival
III:5	+	2022	29	M	*N*	Normal	IA	IA	Polycystic kidney with calculus	Survival
III:6	+	2022	16	M	*N*	Normal	IA	IA	Polycystic kidney	Survival
III:7	+	2022	29	M	*N*	Normal	Normal	*N*	Normal	Survival
III:8	+	2022	21	F	*N*	Normal	IA	IA	Left kidney with double renal sinuses	Survival
III:9	+	2022	25	M	*N*	Normal	Normal	PKD	Multiple hepatic and renal cysts with stones	Survival
IV:1	_	2022	9	M	*N*	Normal	Normal	*N*	Normal	Survival
IV:2	_	2022	1	F	*N*	Normal	*N*	*N*	Normal	Survival
IV:3	+	2022	6	M	*N*	Normal	*N*	*N*	Normal	Survival
IV:4	+	2022	1	F	*N*	Normal	*N*	*N*	Normal	Survival

*Note:* + positive; −, negative; *N*, no. Among the group members, except for ll:5 with a blood creatinine level of 1120 μM, the physiological levels of other examined parameters were normal. Other parameters included liver and kidney function, electrolytes, blood lipids, myocardial enzyme spectrum, blood sugar, blood uric acid, routine defecation, chest and lung computed tomography, and heart and great vessel color ultrasound findings.

Abbreviations: ICH: intracranial hemorrhage; NA: not available; PKD: polycystic kidney disease; SAH: subarachnoid hemorrhage.

### 3.1 Genetic Analysis of the Clinically Diagnosed ADPKD Cohort

**FIGURE 4:**
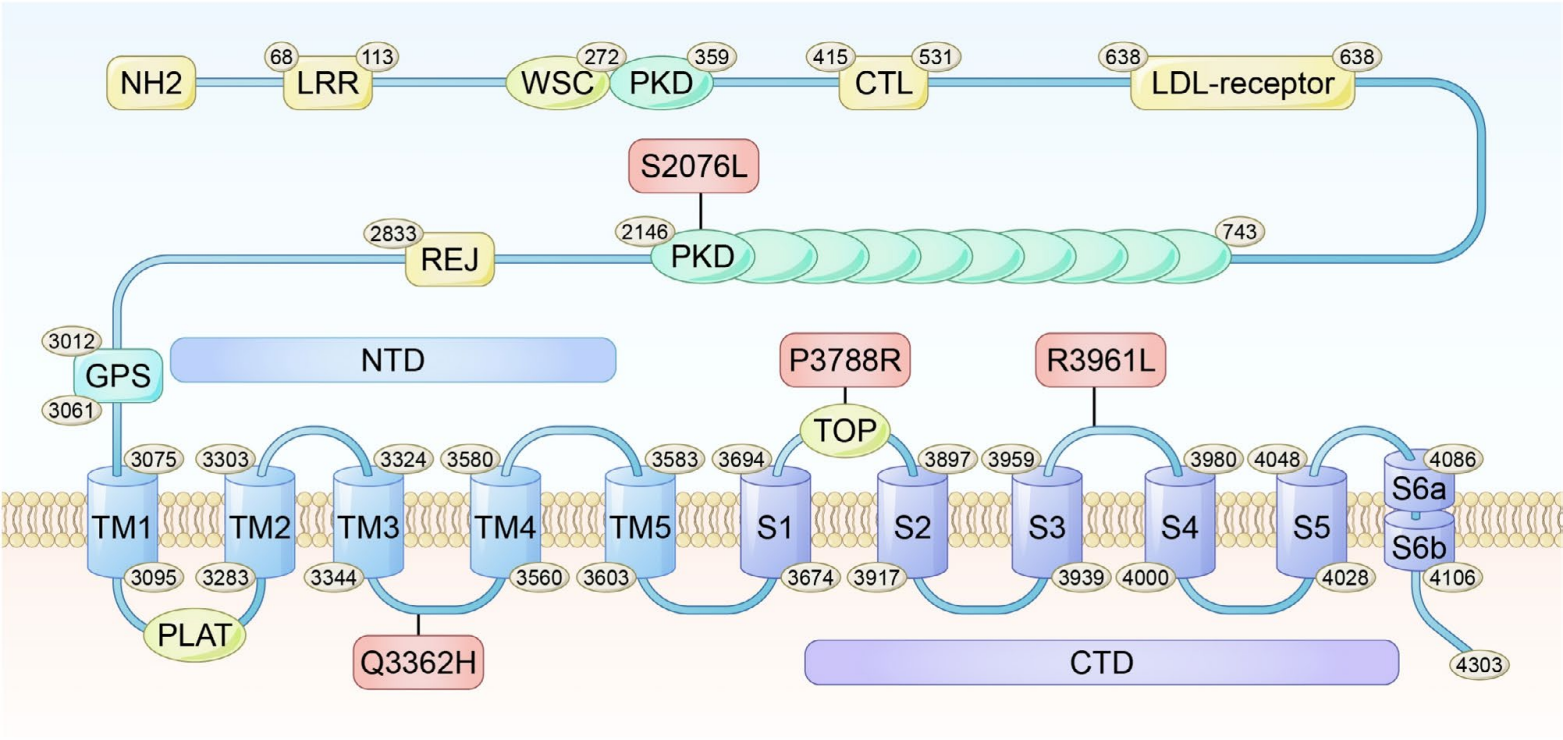
Schematic illustration depicting polycystin‐1 (PC1) and indicating the locations of the *PKD1* mutations identified in the present study. LRR, leucine‐rich repeats; WSC, cell wall integrity and stress response component. LDL, low‐density lipoprotein; REJ, receptor for egg jelly; GPS, G protein‐coupled receptor proteolysis site; PLAT, polycystin‐1, lipoxygenase, alpha‐toxin; NTD, N‐terminal domain (which contains five TMs); TMs, transmembrane helices; TOP, also known as the polycystin domain; CTD, C‐terminal domain (which includes S1 to S6 and the TOP domain).

**FIGURE 5:**
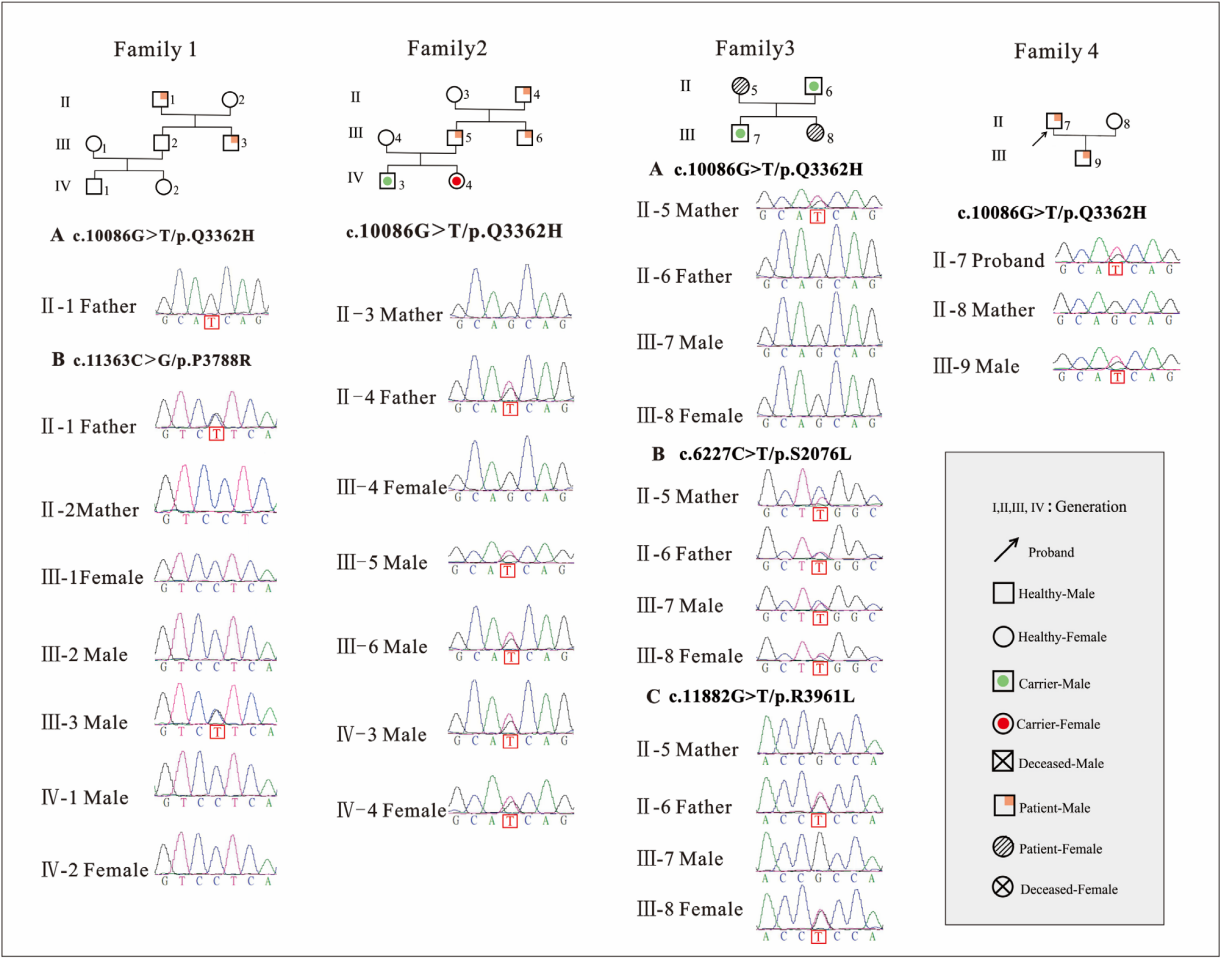
Family sanger sequencing verification. Pedigree and DNA sequencing chromatograms of four families with *PKD1* variants and their corresponding phenotypes.

Four new heterozygous *PKD1* variants were identified for the first time using gene sequencing: c.C11363G/p.P3788R, c.G10086T/p.Q3362H, c.C6227T/p.S2076L, and c.G11882T/p.R3961L (Table 2). The S2076L variant was identified within the PKD repeat domains, whereas the remaining three variants were situated within the transmembrane helix domain, specifically Q3362 in the TM3–TM4 linker and P3788R and R3961L in the S1–S2 linker (Figure 4). No discernible hotspot variants or regions were identified. For family Sanger sequencing verification, see Figure 5.

**TABLE 2:** Mutation information of four types of *PKD1*.

Gene	Chr	AA Change	Exonic Func	GnomAD
*PKD1*	chr16:2142096	NM_001009944:exon40:c.C11363G:p.P3788R	Nonsynonymous SNV	0
*PKD1*	chr16:2147950	NM_001009944:exon31:c.G10086T:p.Q3362H	Nonsynonymous SNV	3.19E‐05
*PKD1*	chr16:2158941	NM_001009944:exon15:c.C6227T:p.S2076L	Nonsynonymous SNV	6.37E‐05
*PKD1*	chr16:2141006	NM_001009944:exon43:c.G11882T:p.R3961L	Nonsynonymous SNV	0

**FIGURE 6:**
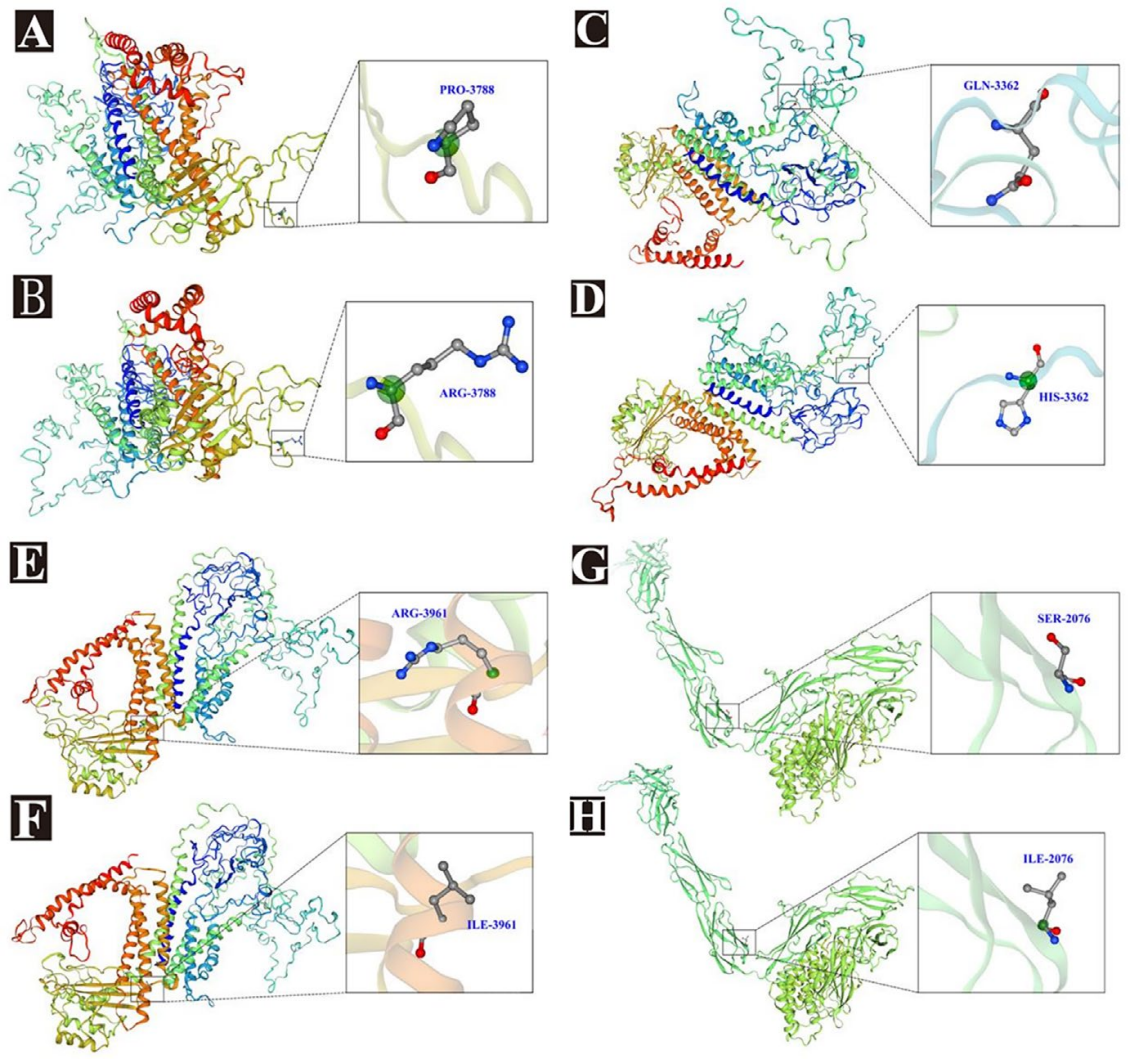
Simulation diagram of the three‐dimensional (3D) protein structure of four *PKD1* gene variants. Swiss‐Model was utilized to predict the 3D structure of the mutant *PKD1*, revealing mutations at positions p.P3788R (A and B), p.Q3362H (C and D), p.R3961L (E and F), and p.S2076L (G and H), which are predicted to disrupt hydrogen bonds with the surrounding residues.

These variants are classified as variants of uncertain significance (PM2) according to the American College of Medical Genetics and Genomics guidelines and are not included in the Human Gene Mutation Database (HGMD), Clinvar, 1000 Genomes, gnomAD databases, or the latest literature on the *PKD1* gene. Three‐dimensional protein structure simulations predicted that these mutations altered the structure of *PKD1* and disrupted hydrogen bonds with surrounding residues (Figure 6).

The first‐generation gene verification of the 21 surviving family members is presented in Table 3.

**TABLE 3:** Distribution of four *PKD1* mutations.

Sample	*PKD1*:c.C11363G	*PKD1*:c.G10086T	*PKD1*:c.C6227T	*PKD1*:c.G11882T
II:1	+	−	*N*	*N*
II:2	−	*N*	*N*	*N*
II:3	*N*	−	*N*	*N*
II:4	*N*	+	*N*	*N*
II:5	*N*	+	+	−
II:6	*N*	−	+	+
II:7	*N*	+	*N*	*N*
II:8	*N*	−	*N*	*N*
III:1	−	*N*	*N*	*N*
III:2	−	*N*	*N*	*N*
III:3	+	*N*	*N*	*N*
III:4	*N*	−	*N*	*N*
III:5	*N*	+	*N*	*N*
III:6	*N*	+	*N*	*N*
III:7	*N*	−	+	−
III:8	*N*	−	+	+
III:9	*N*	+	*N*	*N*
IV:1	−	*N*	*N*	*N*
IV:2	−	*N*	*N*	*N*
IV:3	*N*	+	*N*	*N*
IV:4	*N*	+	*N*	*N*

### 3.2 Impact of *PKD1* Variants on PC1 Function

**FIGURE 7:**
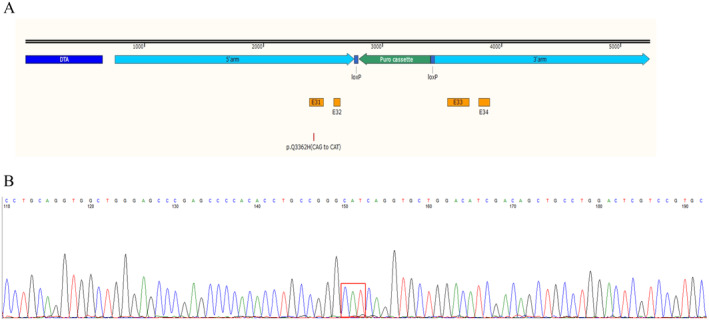
CRISPR/Cas9‐mediated *PKD1* editing in HEK293T. (A) Schematic representation of the CRISPR target site in the human PKD1 Q3362H; DTA: diphtheria toxin A; (B) sequencing chromatogram demonstrating CRISPR/Cas9‐mediated genome editing of PKD1 Q3362H.

**FIGURE 8:**
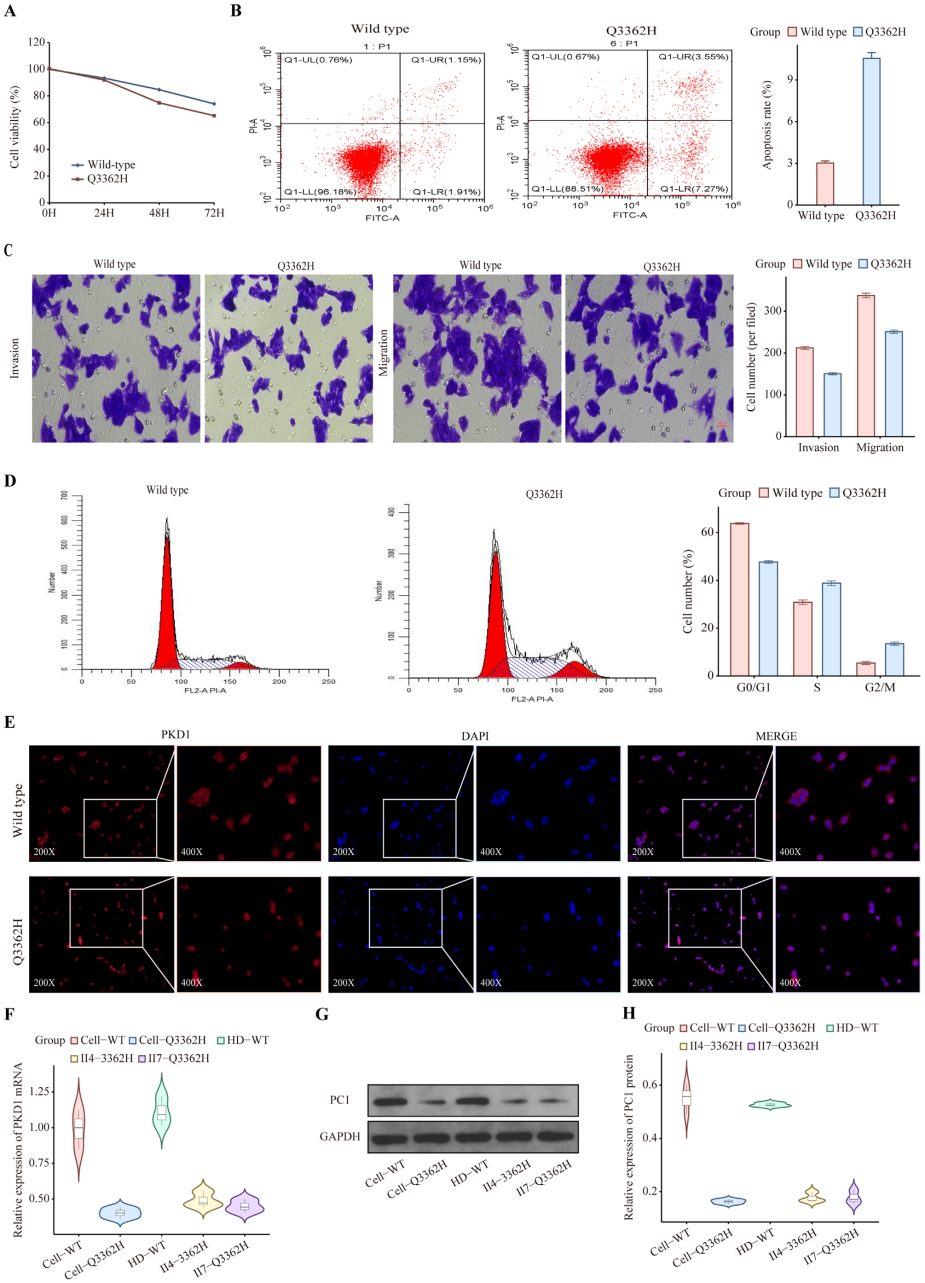
Impact of *PKD1* variants on cellular function. (A) Cell counting kit‐8 (CCK‐8) results showing reduced proliferation in Q3362H mutant HEK‐293T cells compared to controls; (B) Flow cytometric detection showing an increase in the number of apoptotic cells in Q3362H mutant HEK‐293T cells compared to controls; (C) Transwell assay revealing reduced cell migration and invasion in Q3362H HEK‐293T cells relative to controls; (D) Flow cytometry detection of cell cycle showing variation in HEK‐293T cells carrying Q3362H mutation compared to controls; (E) *PKD1* subcellular localization showing reduced expression and nuclear translocation in Q3362H HEK‐293T cells compared to controls; (F) Boxplot of *PKD1* gene expression difference detected by qPCR in Q3362H HEK‐293T cells compared to controls; (G and H) Western blot detection and quantification of PC1 protein expression differences in Q3362H mutant compared to wild‐type conditions.

To explore the effect of the c.G10086T/p. Q3362H *PKD1* mutation on PC1 function, we constructed a c.10086G>T (p.Q3362H) *PKD1* homozygous mutant HEK293T cell line using CRISPR/Cas9 technology (Figure 7). Cells harboring the mutation proliferated at significantly slower rates than the WT, and the proportion of viable mutant cells decreased compared to the WT over 48 h (Figure 8A), with more cells undergoing apoptosis (Figure 8B). Cell cycle analysis (Figure 8D) indicated that the majority of *PKD1* Q3362H mutant cells were in the S and G2/M phases, displaying modified cell cycle properties as well as invasion and migration abilities(Figure 8C) compared to wild‐type cells. Based on 4′,6‐diamidino‐2‐phenylindole (DAPI) and antibody co‐staining, immunofluorescence (Figure 8E) showed that wild‐type *PKD1* primarily resulted in nuclear PC1 expression, whereas *PKD1* Q3362H led to the enrichment of PC1 in the cytoplasm.

RNA was extracted and used to detect the expression level of *PKD1* mRNA using reverse transcription‐quantitative polymerase chain reaction (RT‐qPCR) in both wild‐type (WT) and mutant cells, which demonstrated a significant reduction in *PKD1* levels in the mutant cell line (Figure 8F). Comparing WT HEK293T cells with mutant cells or patient tissues, western blotting also demonstrated significantly decreased PC1 protein expression in HEK293T Q3362H mutant cells (*p* < 0.05), consistent with the detection of skin blood vessels in patients carrying this mutation (Figure 8G,H).

## 4 Discussion

In this study, we identified a novel *PKD1* mutation (c.G10086T/p.Q3362H) that co‐segregates with concurrent ADPKD and IAs in a Chinese family. Through comprehensive functional analyses, we demonstrated that this mutation substantially affects PC1 protein expression and cellular function, providing new insights into the molecular mechanisms underlying ADPKD‐associated vascular complications.

The high prevalence of IAs in our family cohort (38.1%) significantly exceeds the reported 4%–11.5% occurrence in general ADPKD populations [8, 37]. This observation, combined with complete co‐segregation of the mutation with the disease phenotype, indicates that c.G10086T represents a high‐risk variant for IA development. Our findings align with recent studies demonstrating that specific *PKD1* mutations can influence vascular complications in ADPKD [38, 39].

Our functional studies revealed alterations in cellular function caused by the Q3362H mutation, including reduced *PKD1* mRNA and PC1 protein expression, altered PC1 subcellular localization, impaired cell proliferation, and modified cell migration capabilities. These findings are consistent with recent work showing that PC1 dysfunction can lead to multiple cellular defects [40]. The predominant cytoplasmic localization of mutant PC1 we observed aligns with findings regarding the critical role of PC1's C‐terminal tail in protein trafficking and cellular localization [41].

The vascular manifestations in our family support previous observations that *PKD1* mutations affect vascular smooth muscle cells and elastic arteries [42]. Our findings extend earlier work showing that polycystin dysfunction alters calcium signaling in vascular smooth muscle cells [43]. The enhanced cell migration and compromised vascular integrity we observed in mutant cells provide mechanistic insights into how *PKD1* mutations may promote IA formation [44, 45].

The identification of this high‐risk variant has important clinical implications. First, it supports the role of genetic testing in risk stratification for ADPKD patients [46]. Second, it suggests the need for more intensive vascular screening in carriers of high‐risk *PKD1* variants [47]. Third, it provides potential molecular targets for developing targeted therapies [48]. Recent advances in therapeutic approaches, including targeted oligonucleotides and precision medicine strategies, suggest promising directions for mutation‐specific treatments [49].

Several limitations of our study warrant discussion. First, the findings are based on a single family, necessitating validation in larger cohorts. Second, the long‐term natural history of IA development in mutation carriers remains to be determined. Third, the complete spectrum of vascular complications associated with this mutation requires further investigation.

Future research directions should include multicenter studies to validate the association between c.G10086T and IA risk, the development of targeted screening protocols for high‐risk variant carriers, and the investigation of potential therapeutic approaches based on the identified molecular mechanisms [50]. Additionally, the exploration of potential protective factors in unaffected mutation carriers may provide insights into disease modification strategies.

In conclusion, the *PKD1*:c.G10086T mutation represents a novel genetic risk factor for IA development in ADPKD. Our findings not only expand the spectrum of known *PKD1* mutations but also provide new insights into the genetic basis of ADPKD‐associated vascular complications. This work supports the development of genotype‐based approaches to risk stratification and management in ADPKD.

## Author Contributions

**Lili Gao:** investigation, writing – original draft, methodology, validation. **Min Lin:** writing – original draft, investigation, conceptualization. **Chenghan Wu:** writing – review and editing, methodology, funding acquisition, project administration, supervision, writing – original draft. **Yuansheng Liao:** conceptualization, methodology, visualization, formal analysis. **Zuopeng Lin:** formal analysis, visualization, methodology, writing – original draft. **Xiaohua Yan:** investigation, conceptualization, funding acquisition, software. **Sheng Lin:** investigation, validation, visualization, software. **Yinzhou Wang:** methodology, validation, conceptualization. **Jing Chen:** conceptualization, investigation, validation, formal analysis. **Zhaocong Zheng:** methodology, validation, software, formal analysis. **Jushan Lin:** methodology, resources, data curation. **Sheng Zhang:** conceptualization, investigation, formal analysis. **Jianhua Guan:** validation, visualization, conceptualization. **Yan Qiu:** validation, visualization, software. **Jilian Liao:** data curation, software, validation. **Lihua Wu:** methodology, visualization, resources.

## Conflicts of Interest

The authors declare no conflicts of interest.

## Supporting information


Data S1.

## Data Availability

The data that support the findings of this study are available from the corresponding author upon reasonable request.
