# Supplementary material for: Novel PKD1 Mutation (c.G10086T) Drives High Intracranial Aneurysm Risk in Autosomal Dominant Polycystic Kidney Disease
Source: Eur J Neurol. 2025 Feb 20;32(2):e70086. doi: 10.1111/ene.70086 (PMC11840425; doi:10.1111/ene.70086)
Supplement: Supplementary file 1 — Data S1. [file ENE-32-e70086-s001.doc]

**Novel *PKD1* Mutation (c.G10086T) Drives High Intracranial Aneurysm Risk in Autosomal Dominant Polycystic Kidney Disease**

**Author: Chenghan Wu**

**Supplementary Material**

**1. Family medical history information**

The proband (II:7), a 47-year-old male, was admitted to the emergency department on May 31, 2017, due to a sudden onset of headache, nausea, and vomiting. His blood pressure was recorded at 195/89 mmHg. A head computed tomography (CT) scan revealed a patchy, high-density shadow on the falx cerebri, measuring approximately 3.2×2.8 cm, accompanied by extensive subarachnoid hemorrhage (Figure 1A). A brain CT angiography (CTA) indicated a fusiform protrusion in the A2 segment of the left anterior cerebral artery, approximately 1.1×0.8 mm in size (Figure 1B, C). The diagnosis was a ruptured left anterior cerebral aneurysm and subarachnoid hemorrhage. An emergency craniotomy revealed a fusiform aneurysm measuring about 1.2×1.0 mm, in the A2 segment of the left anterior cerebral artery and was treated with a titanium clip. Postoperative CTA review confirmed proper clipping of the aneurism (Figure 1D). Further investigations, including an abdominal color Doppler ultrasound, detected bilaterally misshapen kidneys with incomplete capsules and multiple cystic anechoic areas of varying sizes—the largest on the right measuring 44×36 mm and on the left 72×61 mm (Figure 1E)—as well as liver cysts, the largest being 38×34 mm (Figure 1F), and splenomegaly. A cardiac and major vessel color Doppler ultrasound showed no abnormalities. Genetic testing revealed a *PDK1* mutation, which is consistent with ADPKD diagnosis.

**Family history**

The mother died at 31 years of age from symptoms suggestive of a cerebrovascular event but was not hospitalized or examined. The father died at 61 years after experiencing a headache, and his death was suspected to be related to cerebrovascular disease. He had three brothers and one sister; the third brother died at 1 year of age from unknown causes.

II:4, the proband was 53-year-old second brother, presented with a sudden headache, left limb weakness, and a blood pressure of 155/110 mmHg on December 7, 2021. Head CT displayed cerebral hemorrhage in the right basal ganglia area. CTA and whole-brain blood vessel digital subtraction angiography (DSA) showed right anterior cerebral artery aneurysm and middle cerebral artery aneurysm, and abdominal imaging demonstrated polycystic changes in the kidneys and liver, as well as splenomegaly (Figure 2A–H).

II:5, the proband’s 50-year-old elder sister with a history of drinking was hospitalized on January 18, 2009, with persistent, severe headache, nausea, and vomiting following alcohol consumption. Her blood pressure was 202/100 mm Hg. Imaging revealed a subarachnoid hemorrhage and bilateral middle cerebral artery aneurysms measuring 2×3 and 2×4 mm, respectively, which were treated with coil embolization, leading to good recovery. However, she experienced recurrence on March 25, 2015. Subsequent imaging showed high-density metal artifacts in the M1 segment of the bilateral middle cerebral arteries, with a new 3×5 mm aneurysm and substantial subarachnoid hemorrhage adjacent to the previously treated site. Original and new aneurysms were clamped using titanium clips. Abdominal color Doppler ultrasound and CT examination showed multiple renal cysts in both kidneys and the liver, splenomegaly, kidney stones, and an elevated blood creatinine level of 222 µM. In August 2018, she developed chronic renal failure with blood creatinine levels of 1120 µM. who require biweekly hemodialysis.

The patient’s family history was consistent with a diagnosis of ADPKD. After a comprehensive physical examination, cerebral aneurysms with polycystic kidneys and livers, and splenomegaly were confirmed in most adult family members (Figure 3). No disease phenotypes were observed among the fourth-generation family members, suggesting that the lack of pathological changes may have been due to their young age.

**2. Pathogenic gene sequencing and related experimental methods**

Considering the clinical and genetic diversity of ADPKDs and the complexity of the *PKD1* gene, a comprehensive genetic analysis workflow was established, incorporating whole-exome sequencing (WES), whole-genome sequencing WGS, RNA-seq, targeted Oxford Nanopore Technologies (Oxford, UK)(ONT) sequencing, and *in silico* analysis of WGS-targeted genome-wide variants. WES was applied to target coding regions. Third-generation targeted sequencing was used to detect mutations in *PKD1* and *PKD2*. RNA-seq and *in silico* analyses were performed to validate and predict the functional consequences of the mutations. All WGS- and WES-detected variants with suspected pathogenicity were confirmed using Sanger sequencing.

1. Genomic DNA extraction, library preparation, whole exome and whole genome sequencing

Peripheral blood samples were collected from each patient and stored in ethylenediamine tetraacetic acid tubes. Exome capture was performed using a SureSelect Human All Exons V6 kit (Agilent Technologies, Inc., Santa Clara, CA, USA) according to the manufacturer’s protocol. The WES and WGS libraries were sequenced using the Novaseq 6000 platform (Illumina, Inc., San Diego, CA) at the Wuhan Kindstar Global Gene Technology Center (Kindstar, Wuhan, China). Each sample was sequenced to a mean depth of 100× coverage for WES and 30× coverage for WGS, to achieve high sensitivity and accuracy for mutation detection.

2) Third-generation targeted sequencing

Multiplex polymerase chain reaction (PCR) amplification was performed using primers specific to the exon regions of *PKD1* and *PKD2*. After library construction, a Nanopore sequencer (ONT) was used for high-throughput sequencing.

3) RNA extraction, library construction, and RNA-Seq

Total RNA was extracted from each sample using TRIzol (Life Technologies, Carlsbad, CA, USA), and cDNA libraries were constructed using the NEBNext Ultra RNA Library Prep Kit for Illumina (NEB, E7530) and NEBNext Multiplex Oligos for Illumina (NEB, E7500) following the standard protocol. The constructed cDNA libraries were sequenced on an Illumina novaseq6000 sequencing platform.

4) WES, WGS and targeted sequencing data analysis

Raw image files were processed using Illumina base-calling software with default parameters, and the sequences for each individual were generated as 150 bp paired-end reads. Paired-end reads were aligned to the human reference genome (GRCh37/HG19) using the Burrows-Wheeler Aligner (BWA, v0.7.17. The analysis pipeline included strict data quality control (QC) steps to clean and map the data, and for variant calling. The Genome Analysis Toolkit (GATK, v4.1.1.0) was used for insertion/deletion realignment, quality score recalibration, and variant identification, with duplicate read removal performed using Picard tools (v4.1.1.0). ANNOVAR (v201804) was used to annotate mutations.

After sequence alignment and variant calling, synonymous variants, intronic variants not located in the exon/intron boundaries, and variants with a minor allelic frequency (MAF) ≥ 1% in the 1000 Genomes Project, dbSNP database, and Exome Aggregation Consortium (ExAC) database were removed from further analysis. NGS reads were visualized using an integrated genomic viewer (IGV).

5) Transcriptome data analysis

Low-quality reads, such as adaptor reads, unknown nucleotides > 5%, or Q20 < 20% (percentage of sequences with sequencing error rates < 1%) were removed using SOAPnuke (v2.1.0). The resulting clean reads were mapped to the human 19 genome using Hisat 2 (v2.2.1). Aligned reads in BAM/SAM(BAM（Binary Alignment/Map）,SAM（Sequence Alignment/Map）) format were further scrutinized to remove potential duplicates. Gene expression levels were estimated employing fragments per kilobase of exon per million fragments mapped (FPKM) values using RSEM software (v1.3.1). DESeq2 (v1.22.2) and Q-values were used to identify differentially expressed genes. Subsequently, differences in gene abundance between samples were calculated based on the ratio of FPKM values. Subsequent analyses were focused on genes with a log_2_ ratio ≥2 and an FDR significance score of <0.01.

1. Sanger sequencing

The targeted variant in *PKD1* was amplified via polymerase chain reaction using ProFlex Base (Applied Biosystems, Singapore). All samples were sequenced using independent PCR. Specific primers flanking the mutation sites are listed in Additional File [1](https://www.ncbi.nlm.nih.gov/pmc/articles/PMC9463787/#MOESM1) (Table S1). The mutant variant was confirmed using an Applied Biosystems 3730XL (Applied Biosystems) in patients and controls.

7）Computational modeling

To evaluate the pathogenicity of candidate variants, the structure of *PKD1* was modeled using SWISS-MODEL(<https://swissmodel.expasy.org/>) to predict the effect of missense mutations on protein structure. PyMOL 2.3 software was used for three-dimensional protein structure visualization and analysis.

8）Generation of 293T cell lines with *PKD1* Q3362H mutation using CRISPR/Cas9 nucleases

Using the CRISPR/Cas9 targeting principle and the CRISPR online design tool available at http://crispr.mit.edu/, two sgRNA sequences with higher scores targeting the 31st exon of *PKD1* were selected: gRNA-A1 (complementary to the reverse strand of the gene) with the sequence CGAGCAACCTGCTCCCGGG-TGG and gRNA-B1 (complementary to the reverse strand of the gene) with the sequence CTTCCGAGCAACCTGCTCC-CGG. A Donor vector was constructed with 5' arm/loxP/Puro cassette/loxP/3' arm. The Neon transfection system (Invitrogen) was used to electroporate CRISPR RNP, gRNA, and donor vectors into 293T cells, with a single 30 ms pulse at 950 V. Following a 7-day culture, the remaining cells were transferred into a 96-well plate at a density of 0.5 cells per well in order to establish a monoclonal cell population. Subsequently, the isolated monoclonal genomic DNA was sequenced using Sanger sequencing to verify the existence of the edited *PKD1* alleles.

9）CCK8

HEK293T cells in the logarithmic growth phase were seeded into a 96-well plate at a cell density of 5 × 10^4^ cells/mL. Cell proliferation was measured using the CCK-8 assay kit (Dojindo, Japan). A total of 1000 cells were seeded in five replicate wells of a 96-well plate in a medium supplemented with 10% FBS, with a volume of 100 μL per well. The absorbance values were measured at 450 nm for each well in a 96-well plate after 24, 48, and 72 h of seeding using an automatic plate reader（BIOBASE, Japan）.

10）Cell apoptosis experiment

The cells were cultured for various durations before being collected and washed with phosphate-buffered saline (PBS) followed by centrifugation at 800 × g for 5 min. The cells were then washed with diluted binding buffer and adjusted to a concentration of 2 × 10^5^ cells/ml. FITC Annexin V (Keji Biotech, Jiangsu, China) and propidium iodide (PI) were added to each tube, mixed gently, and incubated at room temperature in the dark for 15 min. Subsequently, the samples were analyzed using flow cytometry within 1 h.

11）Cell cycle analysis

The cells were collected at various time points during culture, washed with PBS, and centrifuged at 800 × g for 5 min. The resulting cell pellet was resuspended in a solution containing 2 ×10^6^ cells/mL, followed by the slow addition of 700 μL of a mixture of pre-cooled 80% ethanol and 1 mg/mL RNase. Subsequently, 10 μL（400μg/ml）of PI was added to the suspension, which was then incubated in the dark at 4 ℃ for 30 min. The samples were analyzed using flow cytometry (BECKMAN, USA).

12）Transwell assay

A transwell assay was performed to assess the migration and invasion abilities of HEK293T cells. Transwell chamber inserts (FALCON, 353097) (Corning, U.S.A.) with or without Matrigel (Corning, 356234) were used for the invasion and migration assays, respectively, according to the manufacturer’s protocol. The transfected cells were seeded into the upper chamber at a density of 1 ×10^4^ cells in 200 µl Dulbecco’s Modified Eagle’s medium (Gibco). Medium containing 10% FBS was added to the lower chamber. The cells were allowed to migrate for 36 h and then invade for 48 h. Cells that migrated or invaded the bottom of the inserts were stained with crystal violet and counted under a microscope. Experiments were performed in triplicate and repeated at least three times. Cells were counted in three randomly chosen fields under a microscope at 200× and 400× magnification, and the average number of cells per field was calculated using ImageJ software (NCBI,...（National Institutes of Health, Bethesda, MD）.

13）Immunofluorescence

After transfection for 48 h, cells on the slides were fixed with 4% paraformaldehyde for 20 min, and then incubated in permeabilizing solution (0.02% Triton X-100 in PBS) for 15 min and blocking buffer (5% goat serum in PBS) for 1 h. The cells were then incubated with anti-Flag antibody (Cell Signaling Technology, 8146S) at 4 °C overnight. On the second day, the cells were incubated with CoraLite488-conjugated goat anti-mouse IgG(H+L) (1:400, SA00013-1, Proteintech) and anti-PC1 antibodies (Abcam, ab235963) for 1 h to visualize PC1-Flag staining. 4',6-diamidino-2-phenylindole (DAPI) Fluoromount-G (Southern Biotech) was used to label the DNA. Finally, cells were imaged using a fluorescence microscope (Leica, Germany).

14）Reverse transcription-quantitative polymerase chain reaction (RT-qPCR)

Gene expression levels were assessed using RT-qPCR. RNA was isolated from cells using the RNeasy system (Qiagen, Germany) and the target genes were amplified using specific primers as detailed in Table S1. The RT-qPCR methodology used the HiScript® II Q Select RT SuperMix for qPCR (VAZYME, R233) and 2*Q3 SYBR qPCR Master Mix (TOLOBIO, 22204) as reagents. All reactions were performed in at least three technical replicates. The relative expression level was calculated using 2^−△△Ct^ normalized to endogenous GAPDH expression. Data are presented as the mean*±*standard deviation, with statistical significance determined by *P* < 0.05, using R statistical analysis.

Table S1. Sequences of real-time polymerase chain reaction primers

| Name | Primer | Sequence | Size |
| --- | --- | --- | --- |
| Homo *GAPDH* | Forward | TCAAGAAGGTGGTGAAGCAGG | 115 bp |
|  | Reverse | TCAAAGGTGGAGGAGTGGGT |  |
| Homo *PKD1* | Forward | TCTGAGGAACCTGAGCCCTA | 193 bp |
|  | Reverse | AGTGGCTGGAGAGGTTCAGA |  |

15）Western blotting

293T cells were lysed using RIPA lysis solution, and proteins resolved on a sodium dodecyl sulfate-polyacrylamide gel electrophoresis (SDS-PAGE) gel were transferred onto PVDF membranes. The membranes were blocked in blocking solution, sequentially incubated with PC1 primary antibody (Affinity, Suzhou, China) and It's HRP-conjugated secondary antibodies. The expression of the target protein was detected using chemiluminescence after washing the membranes with tris buffered saline containing Tween-20 (TBST).

Proteins from the different groups were separated on 10% or 12% SDS-PAGE and transferred onto nitrocellulose membranes. The membranes were washed with TBS-T and blocked in a 5% powdered milk solution in TBS-T for 1 h. After washing with TBS-T, the membranes were probed separately with antibodies against GAPDH (Proteintech, 60004-1-Ig) or PC1(Affinity, AF6443) for 1 h. After washing with TBS-T, the membranes were incubated with a secondary horseradish peroxidase-labelled anti-mouse antibody (Proteintech, SA00001-1) or anti-rabbit antibody (Beyotime, A02084030-05) for 1 h at room temperature. Proteins were visualized using ECL Plus Western Blotting Substrate (Thermo Fisher Scientific) according to the manufacturer’s instructions.
